# Comparative Efficacy of Preoperative, Postoperative, and Perioperative Treatments for Resectable Colorectal Liver Metastases: A Network Meta-Analysis

**DOI:** 10.3389/fphar.2019.01052

**Published:** 2019-09-18

**Authors:** Chao Huang, Jun Huang, Hongliang Luo, Zhen Zong, Zhengming Zhu

**Affiliations:** Department of Gastrointestinal Surgery, The Second Affiliated Hospital of Nanchang University, Nanchang, China

**Keywords:** colorectal liver metastasis, hepatic arterial infusion, chemotherapy, overall survival, disease-free survival, network meta-analysis

## Abstract

**Background:** Several treatment strategies are used for management of resectable colorectal liver metastases. We performed a Bayesian network meta-analysis to compare preoperative, postoperative, or perioperative treatments, identifying the optimal approach.

**Methods:** We searched reports of randomized controlled trials through the relevant databases. The primary outcome criterion was overall survival (OS). The secondary outcome measure was disease-free survival (DFS). We calculated the hazard ratio (HR) with the 95% credible interval (Crl) of the time-to-event data. Rank probabilities were evaluated by the probability of treatment rankings. Multiple treatment comparisons based on a Bayesian network integrated the efficacy of all included approaches.

**Results:** Twenty-two eligible randomized controlled trials with 6,115 patients were included in the network meta-analysis. One treatment that resulted in a significant improvement in OS compared with surgery alone was hepatic arterial infusion (HAI) plus postoperative chemotherapy (CT) [HR = 0.74 with 95% Crl: (0.60, 0.94)]. With regard to the secondary outcome measure, three approaches that led to a significant improvement in DFS compared with surgery alone were HAI plus postoperative CT [HR = 1.44 with 95% Crl: (1.19, 1.75)], postoperative CT [HR = 1.14 with 95% Crl: (1.01, 1.29)], preoperative hepatic and regional arterial chemotherapy (PHRAC) plus preoperative CT [HR = 1.41 with 95% Crl: (1.03, 1.89)]. According to the results for the rank probabilities of the 11 treatments, the combination of HAI and bevacizumab plus postoperative CT showed the highest probability of benefitting OS, and PHRAC plus preoperative CT was most likely to benefit DFS.

**Conclusions:** The combination of HAI and bevacizumab plus postoperative CT exhibited the greatest odds of being the most effective treatment for improving OS, and PHRAC plus preoperative CT exhibited the greatest odds of improving DFS. Further clinical studies are needed and justified.

## Introduction

Over 1.8 million new colorectal cancer (CRC) cases and 881,000 deaths are estimated to occur in 2018, accounting for approximately 1 in 10 cancer cases and deaths; CRC ranks third in terms of incidence but second in terms of mortality (Bray et al., 2018). The liver is the most common organ where distant metastases from CRC occur, and approximately half of CRC patients will develop liver metastases (Leporrier et al., 2006; Manfredi et al., 2006). Liver resection is the best, and possibly curative, treatment for colorectal liver metastasis (CRLM), and 5-year posthepatectomy survival rates is reported to be 45–61% (Tournigand et al., 2004). Unfortunately, approximately 66.7% of patients experience recurrence, *of which 50% occur in the residual liver* (Fong and Salo, 1999; de Jong et al., 2009; D’Angelica et al., 2011). Microscopic residual after surgery is the most likely cause of recurrence. Therefore, combining chemotherapy (CT) with resection of CRLM is of major interest.

Randomized controlled trials (RCTs) *have* provided some indication that postoperative CT administered after hepatectomy either through the hepatic artery (HA) or intravenously can improve the prognosis (Lorenz et al., 1998; Kemeny set al., 1999; Kemeny et al., 2002; Power and Kemeny, 2010). Hepatic arterial infusion (HAI) CT significantly increases disease-free survival (DFS) compared with systemic therapy alone in three of four randomized studies (Lorenz et al., 1998; Kemeny et al., 1999; Lygidakis et al., 2001; Kemeny et al., 2002). Most RCTs have also demonstrated that perioperative adjuvant portal vein infusion (PVI) CT in patients with CRC significantly increases overall survival (OS) and DFS when compared with surgery alone (Beart et al., 1990; Fielding et al., 1992). Previously, published results from the European Organisation for Research and Treatment of Cancer intergroup trial 40983 (EPOC) and meta-analysis showed that the combination of perioperative CT with FOLFOX4 and surgery significantly increases progression-free survival (PFS) and DFS when compared with no systemic treatment in resected patients (Mitry et al., 2006; Nordlinger et al., 2008). Preoperative hepatic and regional arterial chemotherapy (PHRAC) combined with surgical resection can improve the survival rate of patients with advanced CRC by significantly decreasing the incidence of liver metastasis (Xu et al., 2007). Regimen of irinotecan (IRI) combined with fluorouracil (FU) plus leucovorin (LV) provide survival benefits over FU and LV for metastatic colorectal cancer (MCRC) (Saltz et al., 2000), which should be considered as a reference first-line treatment (Douillard et al., 2000). The addition of bevacizumab (BEV) to FU-based combination CT results in a statistically significant and clinically meaningful improvement in survival among patients with MCRC (Hurwitz et al., 2004). The results of an RCT have shown that active specific immunotherapy (ASI) with a Newcastle disease virus (NDV)-infected autologous tumor cell vaccine in colon cancer patients appears to be beneficial for prolonging overall and metastasis-free survival (Schulze et al., 2009).

However, optimum treatment in relation to OS or DFS is still lacking, and some of the treatments have never been compared with each other because of the lack of head-to-head trials and the limitations of traditional meta-analysis methods. Thus, there is still uncertainty regarding which is the best treatment for patients with resectable CRLM. We performed a meta-analysis of RCTs by using network meta-analysis (NMA) as a methodology (Cipriani et al., 2013). The aims of our NMA were to obtain the comparative efficacy of the treatments by summarizing the indirect and direct evidence for comparative DFS and OS under various preoperative, postoperative, and perioperative treatments.

## Methods

Literature screening was performed according to the preferred reporting items for systematic reviews and meta-analyses flow chart (Moher et al., 2009) and the report of the International Society for Pharmacoeconomics and Outcomes Research Task Force on Indirect Treatment Comparisons Good Research Practices (Jansen et al., 2011). Institutional review board approval was not required.

## Search Strategy

The PubMed, Embase, Cochrane Library, and ISI-Web of Science databases were searched systematically for articles published between 1950 and 2018 from October 19, 2018, to November 25, 2018. The following search terms were used in several logical combinations: colorectal neoplasms, colorectal tumor, colorectal carcinoma, colorectal cancer, liver neoplasms, hepatic neoplasm, hepatic cancer, liver cancer, neoplasm metastasis, metastases, neoadjuvant therapy, neoadjuvant treatment, adjuvant CT, perioperative period, postoperative period, preoperative period, surgical procedures, operative procedure, RCT, randomized, and randomly. We also carefully read the references of relevant studies.

## Study Selection

The criteria for eligibility were as follows, considering that the RCTs compared at least two of the following treatment strategies (CRLM is available and has been studied in RCTs): HAI, PVI, cetuximab (CET) plus perioperative CT，PHRAC plus preoperative CT, HAI plus postoperative CT, perioperative CT, IRI plus postoperative CT, postoperative CT, combination of HAI and BEV plus postoperative CT, ASI, and surgery alone. Additionally, for eligibility, the patients with resectable CRLM should be administered after curative-intent surgery, and the HRs and 95% confidence intervals (CIs) for OS and DFS can be estimated based on the information in the article. Duplicate studies were removed using EndNote version X7.7 (Thomson Reuters). Studies that fulfilled the eligibility criteria were evaluated in full-text form. We exclude studies that are not RCTs and have unavailable data.

## Data Collection and Assessment of Risk of Bias

The data were extracted by two investigators independently using the same standardized collection form. Relevant data were collected, including the first author, the year of publication, country, patient characteristics, treatment strategies, sample size, and outcomes (OS and DFS). Qualitative assessment was accomplished by two reviewers independently, and if there were disagreements, it was discussed with the third reviewer. Qualitative assessment of the articles was conducted using the Cochrane Collaboration tool and the RoB 2.0 tool for assessing the risk of bias in randomized trials (Higgins et al., 2011; Higgins et al., 2016).

## Statistical Analysis

The primary outcome criterion of our NMA was OS, and the secondary outcome measure was DFS. For time-to-event data, treatment effects were assessed as HRs, which take the number and timing of events into consideration. The 95% CIs were used for the direct meta-analysis and Crl for the NMA estimates. Survival data were obtained directly from the articles or estimated using the Kaplan–Meier survival curve reported by Tierney et al. (Tierney et al., 2007).

Heterogeneity was assessed by the Cochran Q test and measured by the I^2^ statistic. Interpretation of the I^2^ values was performed by assigning low, moderate, and high attributes in cases showing values of 0 to 25%, 25 to 50%, and above 75%, respectively (Higgins and Thompson, 2002). Risk of bias was assessed using the dedicated Cochrane tool of Review Manager (RevMan. Version 5.3.Copenhagen: The Nordic Cochrane Centre, The Cochrane Collaboration, 2014). In addition, we also conducted an assessment of the methodological quality of the studies using the RoB 2.0 tool for RCTs (Higgins et al., 2016).

First, we conducted a traditional pairwise meta-analysis with Stata 14.2 (Stata Corp, College Station, TX) for direct comparisons, synthesizing studies that compared the same treatment with a random-effect model. Second, we performed NMA within a Bayesian framework *via* the Markov chain Monte Carlo method in OpenBUGS 3.2.3 software (Caldwell et al., 2005; Lunn et al., 2009). We selected a fixed or random effect based on the deviance information criteria (DIC) and heterogeneity; the residual deviance statistics and DIC were used to evaluate the model fit for the consistent and inconsistent models (Spiegelhalter et al., 2002; Dias et al., 2011). A more complex model will generally exhibit a better fit to the data and will result in smaller residual deviance. Thus, the model with the lowest DIC is preferred. If a model shows the smallest posterior mean residual deviance, heterogeneity, or DIC value, this indicates consistency in the data. The convergence of the models to their posterior distributions was assessed using the Brooks–Gelman–Rubin convergence statistic (Brooks and Gelman, 1998). We ran three chains each with a burn-in of 5,000 iterations and a thinning interval of 10, which was sufficient to ensure convergence as judged by inspection of the chain histories, and then sampled the posterior distributions from further 15,000 iterations of each chain. The Bayesian analysis ranked the treatments and provided the probability of attaining that rank based on the proportion of Markov chain iterations in which the treatment exhibited the highest probability of lowering the risk of mortality.

## Results

### Study Selection and Characteristics

**Figure 1:**
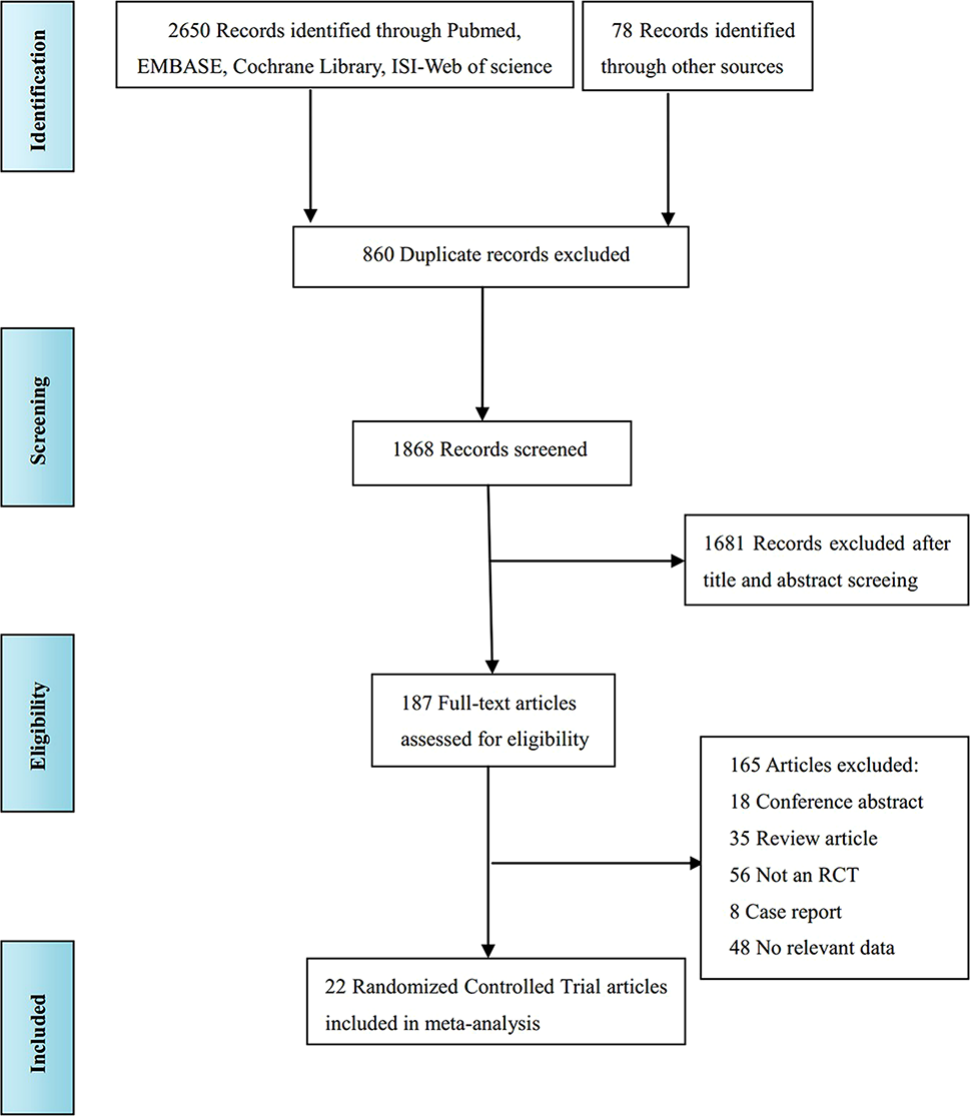
Study flow diagram.

The literature screening process is shown in **Figure 1**. A total of 2,728 records were identified from various databases, including PubMed, Embase, Cochrane Library, ISI-Web of Science, and references; 860 records were excluded because the title showed that they were identical. Among the remaining 1,868 studies, 1,681 were excluded because, according to title and abstract screening, the field of these studies was not relevant. Hence, 187 full-text articles were considered; among these studies, 165 were removed for the following reasons: 18 were conference abstracts, 35 were review articles, 56 were not RCTs, 8 were case reports, and 48 reported unextractable data. Finally, 22 studies (Beart et al., 1990; Wolmark et al., 1990; Lorenz et al., 1998; Kemeny et al., 1999; Rudroff et al., 1999; Tono et al., 2000; Kemeny et al., 2002; Langer et al., 2002; Sadahiro et al., 2004; Kemeny et al., 2006; Portier et al., 2006; Parks et al., 2007; Xu et al., 2007; Laffer et al., 2008; Schulze et al., 2009; Ychou et al., 2009; Kemeny et al., 2011; Bolton et al., 2012; Feng et al., 2012; Nordlinger et al., 2013; Primrose et al., 2014; Hasegawa et al., 2016) were included for quality evaluation and quantitative analysis.

**Figure 2:**
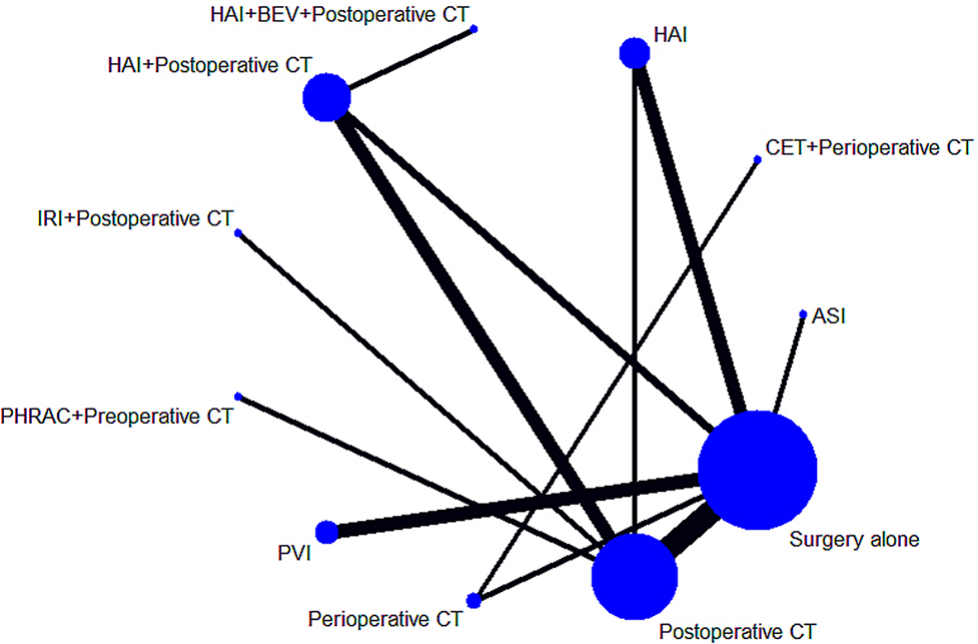
Network of the comparisons for the Bayesian network meta-analysis. The sizes of the nodes and the thicknesses of the edges are weighted according to the number of studies evaluating each treatment and direct comparison, respectively.

The characteristics of the included studies are shown in **Table 1**. Our analysis included 6,115 patients treated in 11 treatments: 2,458 treated with surgery alone, 327 treated with HAI plus postoperative CT, 1,322 treated with postoperative CT, 340 treated with HAI, 937 treated with PVI, 279 treated with perioperative CT, 110 treated with PHRAC plus preoperative CT, 129 treated with CET plus perioperative CT, 153 treated with IRI plus postoperative CT, 35 treated with the combination of HAI and BEV plus postoperative CT, and 25 treated with ASI. **Figure 2** shows the network plot of the comparison of 11 treatments.

**Table 1:** Characteristics of the 22 included studies.

Author	Year	Country	Study Design (Intervention/Control	Sample size	Regimen	**Tumor location (%)**	Median age(years)	Median follow-up (Months)	Outcomes	Conflicts of interest	Risk-of -bias
Kemeny et al.	2002	Australia	HAI+Postoperative CT/Surgery alone	30/45	FUDR 0.1 mg/kg/d, 5-FU 200 mg/m²/d	Colon (53.3%) Rectum (45.3%)	59/62	NR	OS,DFS	NR	Some concerns
Feng et al.	2012	China	HAI+Postoperative CT/Postoperative CT	140/147	OXA 85 mg/m²,FA 200 mg/m²,5-FU 2400 mg/m²	Colon (64.8%) Rectum (35.2%)	64.3/65.2	33.7	OS,DFS	NR	Low
Lorenz et al.	1998	Germany	HAI/Surgery alone	108/111	5-FU 1000 mg/m²/d, FA 200 mg/m²/d	Colon (31.5%) Rectum (34.7%)	61/61	40.8	OS,DFS	NR	Low
Sadahiro et al.	2004	Japan	HAI/Surgery alone	150/155	5-FU 250 mg/d	Colon (100%)	60/60	61.3	OS,DFS	NR	Low
Hasegawa et al.	2016	Japan	Postoperative CT/Surgery alone	88/89	UFT 300 mg/m², LV 75 mg/d Orally	Colon (62.1%) Rectum (37.9%)	62.3/64.4	57.1	OS,DFS	NR	Low
Laffer et al.	2008	Switzerland	PVI/Surgery alone	250/252	5-FU 500 mg/m², mitomycin C 10 mg/m²	Colon (64.3%) Rectum (35.7%)	63.5/64	93.6	OS,DFS	NR	Low
Laffer et al.	2008	Switzerland	Postoperative CT/Surgery alone	251/252	5-FU 500 mg/m², mitomycin C 10 mg/m²	Colon (62.6%) Rectum (37.4%)	63.5/63	93.6	OS,DFS	NR	Low
Nordlinger et al.	2013	France	Perioperative CT/Surgery alone	151/152	OXA 85 mg/m²,FA 200 mg/m²,5-FU 600 mg/m²	Colon (55.4%) Rectum (41.3%)	62/64	102	OS,DFS	Yes	Low
Portier et al.	2006	France	Postoperative CT/Surgery alone	86/85	5-FU 400 mg/m², FA 200 mg/m²	Colon (59.1%) Rectum (40.9%)	NR	87	OS,DFS	NR	Low
Xu et al.	2007	China	PHRAC+Preoperative CT/Postoperative CT	110/112	FUDR 500 mg,OXA 50 mg, DEX 2.5 mg	Colon (54.1%) Rectum (45.9%)	59/60	35	OS,DFS	NR	Low
Langer et al.	2002	Switzerland	Postoperative CT/Surgery alone	52/55	NA	NA	NA	NA	OS,DFS	NA	High
Primrose et al.	2014	UK	CET+Perioperative CT/Perioperative CT	129/128	OXA 130 mg/m²,5-FU 2400 mg/m²,Capecitabine 1000 mg/m²,CET 500 mg/m² orally	NR	63/64	20.7	OS,DFS	Yes	Low
Kemeny et al.	1999	USA	HAI+Postoperative CT/Postoperative CT	74/82	FUDR 0.25 mg/m²/d, DEX 20 mg,LV 200 mg/m², 5-FU 370 mg/m²	NR	59/59	62.7	OS,DFS	NR	Low
Rudroff et al.	1999	Germany	HAI/Surgery alone	14/16	5-FU 800 mg/m², mitomycin C 8 mg/m²	Colon (36.7%) Rectum (63.3%)	58/57	NR	OS,DFS	NR	Some concerns
Tono et al.	2000	Japan	HAI+Postoperative CT/Postoperative CT	9/10	5-FU 500 mg/d, 5-FU 200 mg/d orally	NR	59/61.9	62.2	OS,DFS	NR	Some concerns
Parks et al.	2007	USA	Postoperative CT/Surgery alone	274/518	5-FU-based	NR	63/65	45	OS,DFS	NR	Low
Ychou et al.	2009	Italy	IRI+Postoperative CT/Postoperative CT	153/153	FA 400 mg/m²,5-FU 400 mg/m²,IRI 180 mg/m²	Colon (72.5%) Rectum (27.5%)	63/61	42.4	OS,DFS	NR	Low
Kemeny et al.	2011	USA	HAI+BEV+Postoperative CT/HAI+Postoperative CT	35/38	FUDR, 0.12 mg//m², BEV 5 mg/kg,OXA 85 mg/m²,LV 400 mg/m²,5-FU 2,000 mg/m²	Colon (72.6%) Rectum (27.4%)	NR	NR	OS,DFS	Yes	Low
Bolton et al.	2012	Mongolia	HAI+Postoperative CT/Surgery alone	36/13	FUDR 0.2 mg/kg/d,5-FU 425 mg/m²/d,LV 20 mg/m²/d	NR	61.5/61	66	OS,DFS	NR	Low
Kemeny et al.	2006	USA	HAI/Postoperative CT	68/67	FUDR 0.18 mg/kg, LV 4 mg/m²,DEX 25 mg pump, LV 20 mg/m², FU 425 mg/m²	NR	57/61	NR	OS,DFS	NR	Low
Wolmark et al.	1990	Panama	PVI/Surgery alone	577/581	5-FU 600 mg/m²	NR	NR	60	OS,DFS	NR	Some concerns
Beart et al.	1990	USA	PVI/Surgery alone	110/109	5-FU 500 mg/m²	NR	65/67	66	OS,DFS	NR	Some concerns
Schulze et al.	2009	Germany	ASI/Surgery alone	25/25	Vaccines containing 1×10^7^ irradiated tumor cells infected with 32 HUNDV	Colon (54.0%) Rectum (46.0%)	58.3/59.7	116.1	OS,DFS	NR	Low

HAI=hepatic arterial infusion; CT=chemotherapy; PVI=portal vein infusion; PHRAC=preoperative hepatic and regional arterial chemotherapy; CET=Cetuximab; IRI=irinotecan; BEV=bevacizumab; ASI=active specific immunotherapy; FUDR=floxuridine; 5-FU=fluorouracil; OXA=oxaliplatin; FA=folinic acid; UFT=uracil-tegafur; LV=leucovorin; DEX=dexamethasone; HU=hemagglutinating units; NDV=Newcastle disease virus; NA=not available; NR=not reported; OS=overall survival; DFS=disease-free survival

Low risk of bias: the study is judged to be at low risk of bias for all domains for this result; Some concerns: the study is judged to raise some concerns in at least one domain for this result, but not to be at high risk of bias for any domain; High risk of bias: the study is judged to be at high risk of bias in at least one domain for this result. Or the study is judged to have some concerns for multiple domains in a way that substantially lowers confidence in the result.

### Comparisons of OS

**Figure 3:**
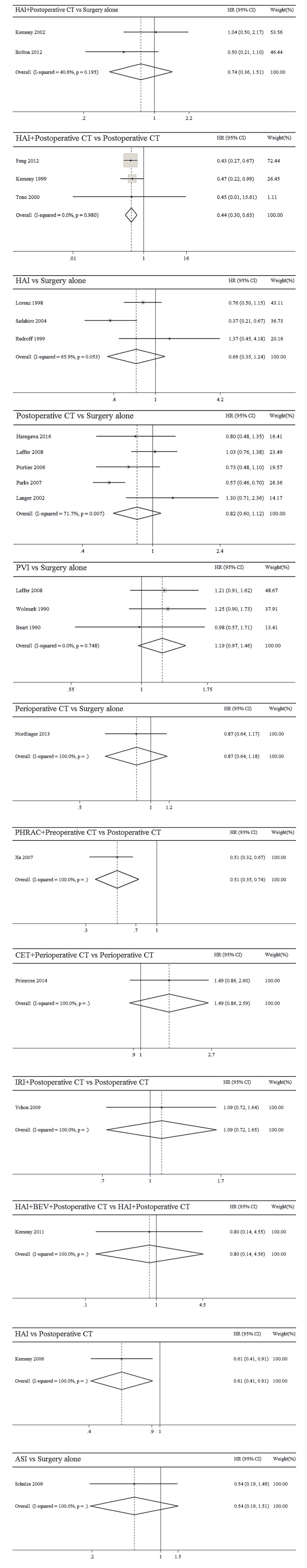
Forest plots of results with pairwise meta-analysis for overall survival.

The results of pairwise meta-analyses are showed in **Table 2**. The heterogeneity and the results of forest plot are summarized in **Figure 3**. Six treatments that were found not to lead to significantly improved OS when compared with surgery alone were HAI plus postoperative CT [HR = 0.74 with 95% CI: (0.36, 1.51)], HAI [HR = 0.66 with 95% CI: (0.35, 1.24)], postoperative CT [HR = 0.82 with 95% CI: (0.60, 1.12)], PVI [HR = 1.19 with 95% CI: (0.97, 1.46)], perioperative CT [HR = 0.87 with 95% CI: (0.64, 1.18)], and ASI [HR = 0.54 with 95% CI: (0.19, 1.51)]. By comparing different treatments, we found statistically significant differences between HAI plus postoperative and postoperative CT [HR = 0.44 with 95% CI: (0.30, 0.65)], PHRAC plus preoperative CT and postoperative CT [HR = 0.51 with 95% CI: (0.35, 0.74)], and HAI and postoperative CT [HR = 0.61 with 95% CI: (0.41, 0.91)].

**Table 2:** Summary of pair-wise meta-analysis results for overall survival.

Comparisons	Results of Pair-Wise Meta-Analysis (HR with 95% CI)	I^2^ (%)
HAI+Postoperative CT vs Surgery alone	0.74 (0.36, 1.51)	40.6
HAI+Postoperative CT vs Postoperative CT	0.44 (0.30, 0.65)	0
HAI vs Surgery alone	0.66 (0.35, 1.24)	65.9
Postoperative CT vs Surgery alone	0.82 (0.60, 1.12)	71.7
PVI vs Surgery alone	1.19 (0.97, 1.46)	0
Perioperative CT vs Surgery alone	0.87 (0.64, 1.18)	–
PHRAC+Preoperative CT vs Postoperative CT	0.51 (0.35, 0.74)	–
CET+Perioperative CT vs Perioperative CT	1.49 (0.86, 2.59)	–
IRI+Postoperative CT vs Postoperative CT	1.09 (0.72, 1.65)	–
HAI+BEV+Postoperative CT vs HAI+Postoperative CT	0.80 (0.14, 4.56)	–
HAI vs Postoperative CT	0.61 (0.41, 0.91)	–
ASI vs Surgery alone	0.54 (0.19, 1.51)	–

**Figure 4:**
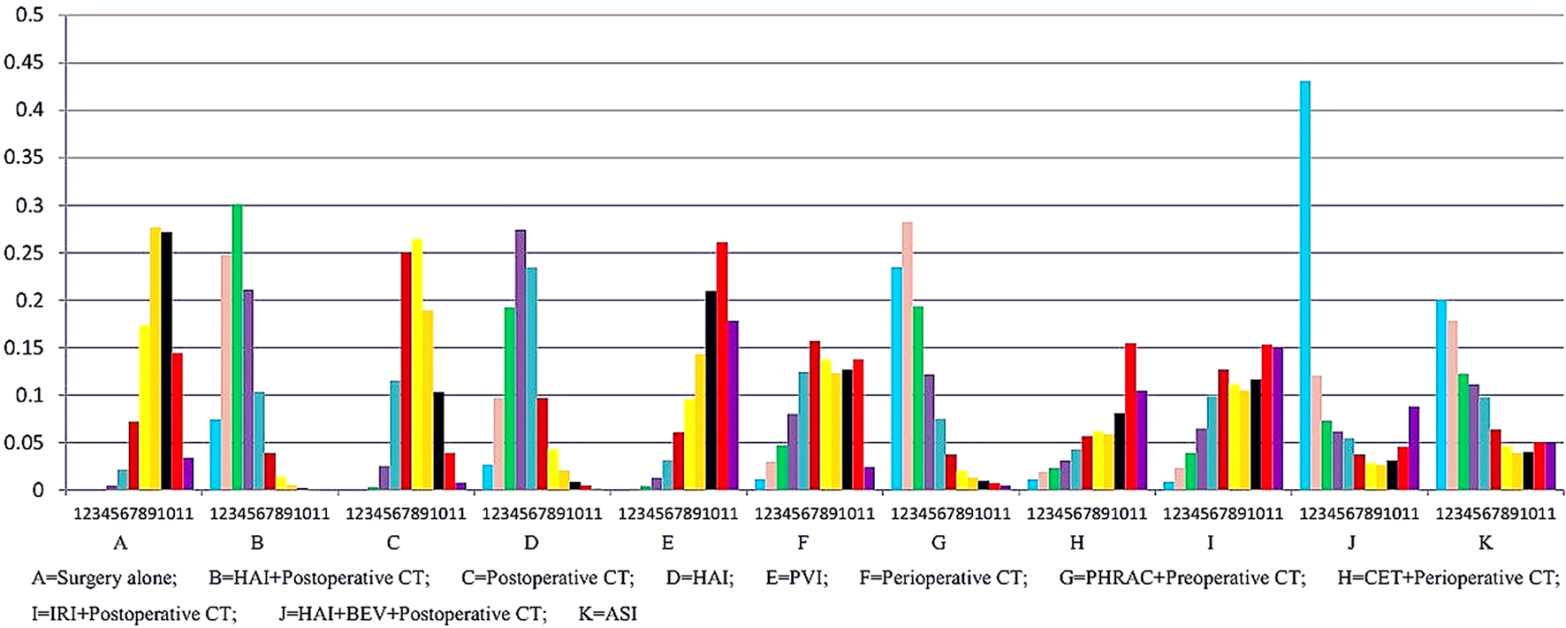
Ranking of treatments in overall survival.

The results of NMA are presented in **Table 3**. In the NMA, one treatment that resulted in a significant improvement in OS compared with surgery alone was HAI plus postoperative CT [HR = 0.74 with 95% Crl: (0.60, 0.94)]. However, perioperative CT and PHRAC plus preoperative CT were not associated with a statistically significant survival advantage compared with surgery alone [HR = 0.94 with 95% Crl: (0.68–1.30); HR = 0.70 with 95% Crl: (0.50–1.03), respectively]. In addition, HAI plus postoperative CT was associated with a statistically significant survival advantage compared with postoperative CT [HR = 1.27 with 95% Crl: (1.02–1.58)]. The results for the rank probabilities of 11 treatments in OS are summarized in **Figure 4**, demonstrating that the combination of HAI and BEV plus postoperative CT resulted in the highest probability of benefitting OS, followed by PHRAC plus preoperative CT, and HAI plus postoperative CT.

**Table 3:** Network meta-analysis of overall survival and disease-free survival.

Surgery alone	**1.44(1.19-1.75)**	**1.14(1.01-1.29)**	1.07(0.90-1.29)	0.91(0.77-1.12)	1.15(0.87-1.51)	**1.41(1.03-1.89)**	0.89(0.60-1.36)	1.20(0.90-1.61)	1.18(0.77-1.80)	1.06(0.70-1.63)
**0.74(0.60-0.94)**	HAI+Postoperative CT	**0.80(0.67-0.95)**	**0.75(0.59-0.96)**	**0.64(0.49-0.84)**	0.81(0.58-1.11)	0.99(0.70-1.36)	0.62(0.40-0.98)	0.84(0.61-1.16)	0.83(0.56-1.21)	0.74(0.47-1.19)
0.94(0.83-1.10)	**1.27(1.02-1.58)**	Postoperative CT	0.94(0.78-1.15)	0.80(0.64-1.03)	1.01(0.75-1.36)	1.24(0.94-1.63)	0.78(0.52-1.21)	1.05(0.81-1.38)	1.04(0.68-1.58)	0.93(0.60-1.45)
0.83(0.68-1.02)	1.09(0.81-1.44)	0.85(0.68-1.05)	HAI	0.86(0.67-1.12)	1.08(0.77-1.48)	1.32(0.93-1.82)	0.83(0.54-1.32)	1.12(0.80-1.55)	1.10(0.70-1.73)	0.99(0.63-1.57)
1.04(0.85-1.29)	1.36(0.91-2.06)	1.14(0.84-1.48)	1.26(0.95-1.65)	PVI	1.16(0.83-1.59)	**1.56(1.08-2.17)**	0.89(0.58-1.41)	1.21(0.86-1.67)	1.19(0.76-1.86)	1.07(0.67-1.69)
0.94(0.68-1.30)	1.37(0.99-1.82)	1.00(0.69-1.40)	1.14(0.78-1.64)	0.87(0.60-1.30)	Perioperative CT	1.23(0.81-1.85)	0.77(0.58-1.04)	1.05(0.70-1.56)	1.03(0.62-1.70)	0.92(0.57-1.54)
0.70(0.50-1.03)	0.95(0.63-1.44)	0.75(0.54-1.03)	0.86(0.58-1.26)	0.65(0.44-1.04)	0.75(0.47-1.26)	PHRAC+Preoperative CT	0.63(0.38-1.05)	0.85(0.58-1.24)	0.83(0.51-1.37)	0.75(0.45-1.25)
1.12(0.68-1.85)	1.13(0.64-2.00)	1.19(0.70-1.96)	1.36(0.80-2.29)	1.04(0.61-1.80)	1.19(0.81-1.73)	1.60(0.86-2.90)	CET+Perioperative CT	1.36(0.84-2.24)	1.34(0.75-2.40)	1.20(0.68-2.17)
0.98(0.68-1.44)	1.32(0.87-2.00)	1.04(0.74-1.45)	1.19(0.80-1.78)	0.91(0.60-1.45)	1.03(0.64-1.73)	1.40(0.87-2.24)	0.86(0.47-1.65)	IRI+Postoperative CT	0.98(0.59-1.63)	0.89(0.53-1.49)
0.67(0.29-1.57)	0.91(0.40-2.05)	0.71(0.31-1.66)	0.82(0.35-1.93)	0.63(0.27-1.51)	0.72(0.29-1.77)	0.96(0.39-2.35)	0.60(0.23-1.61)	0.69(0.28-1.68)	HAI+BEV+Postoperative CT	0.90(0.49-1.63)
0.76(0.45-1.30)	1.03(0.57-1.84)	0.81(0.46-1.39)	0.93(0.52-1.62)	0.71(0.41-1.25)	0.81(0.44-1.53)	1.09(0.57-2.03)	0.68(0.33-1.42)	0.78(0.40-1.46)	1.15(0.42-2.94)	ASI

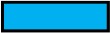
Treatment 
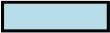
Overall survival, HR (95%Crl) 
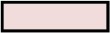
Disease-free survival, HR (95%Crl). Significant results are in bold and underlined.

### Comparisons of Disease-Free Survival

**Figure 5:**
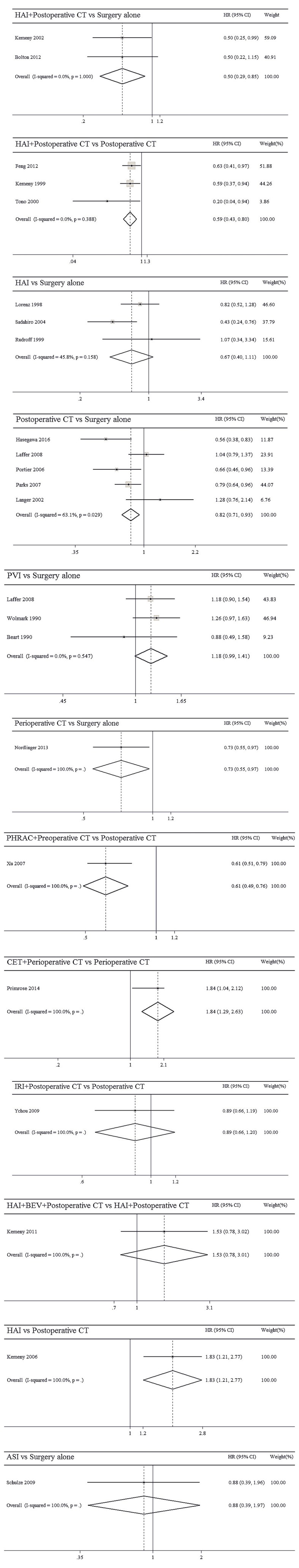
Forest plots of results with pairwise meta-analysis for disease-free survival.

The results of pairwise meta-analyses are presented in **Table 4**. The heterogeneity and the results of forest plot are summarized in **Figure 5**. Compared with surgical resection alone, three approaches for significantly improving DFS were HAI plus postoperative CT [HR = 0.50 with 95% CI: (0.29, 0.85)], postoperative CT [HR = 0.82 with 95% CI: (0.71, 0.93)], and perioperative CT [HR = 0.73 with 95% CI: (0.55, 0.97)]. By comparing different treatments, we found statistically significant differences between HAI plus postoperative and postoperative CT [HR = 0.59 with 95% CI: (0.43, 0.80)], PHRAC plus preoperative CT and postoperative CT [HR = 0.61 with 95% CI: (0.49, 0.76)], CET plus perioperative CT and perioperative CT [HR = 1.84 with 95% CI: (1.29, 2.63)], and HAI and postoperative CT [HR = 1.83 with 95% CI: (1.21, 2.77)].

**Table 4:** Summary of pair-wise meta-analysis results for disease-free survival.

Comparisons	Results of Pair-Wise Meta-Analysis (HR with 95% CI)	I^2^ (%)
HAI+Postoperative CT vs Surgery alone	0.50 (0.29, 0.85)	0
HAI+Postoperative CT vs Postoperative CT	0.59 (0.43, 0.80)	0
HAI vs Surgery alone	0.67 (0.40, 1.11)	45.8
Postoperative CT vs Surgery alone	0.82 (0.71, 0.93)	63.1
PVI vs Surgery alone	1.18 (0.99, 1.41)	0
Perioperative CT vs Surgery alone	0.73 (0.55, 0.97)	–
PHRAC+Preoperative CT vs Postoperative CT	0.61 (0.49, 0.76)	–
CET+Perioperative CT vs Perioperative CT	1.84 (1.29, 2.63)	–
IRI+Postoperative CT vs Postoperative CT	1.09 (0.72, 1.65)	–
HAI+BEV+Postoperative CT vs HAI+Postoperative CT	1.53 (0.78, 3.01)	–
HAI vs Postoperative CT	1.83 (1.21, 2.77)	–
ASI vs Surgery alone	0.88 (0.39, 1.97)	–

**Figure 6:**
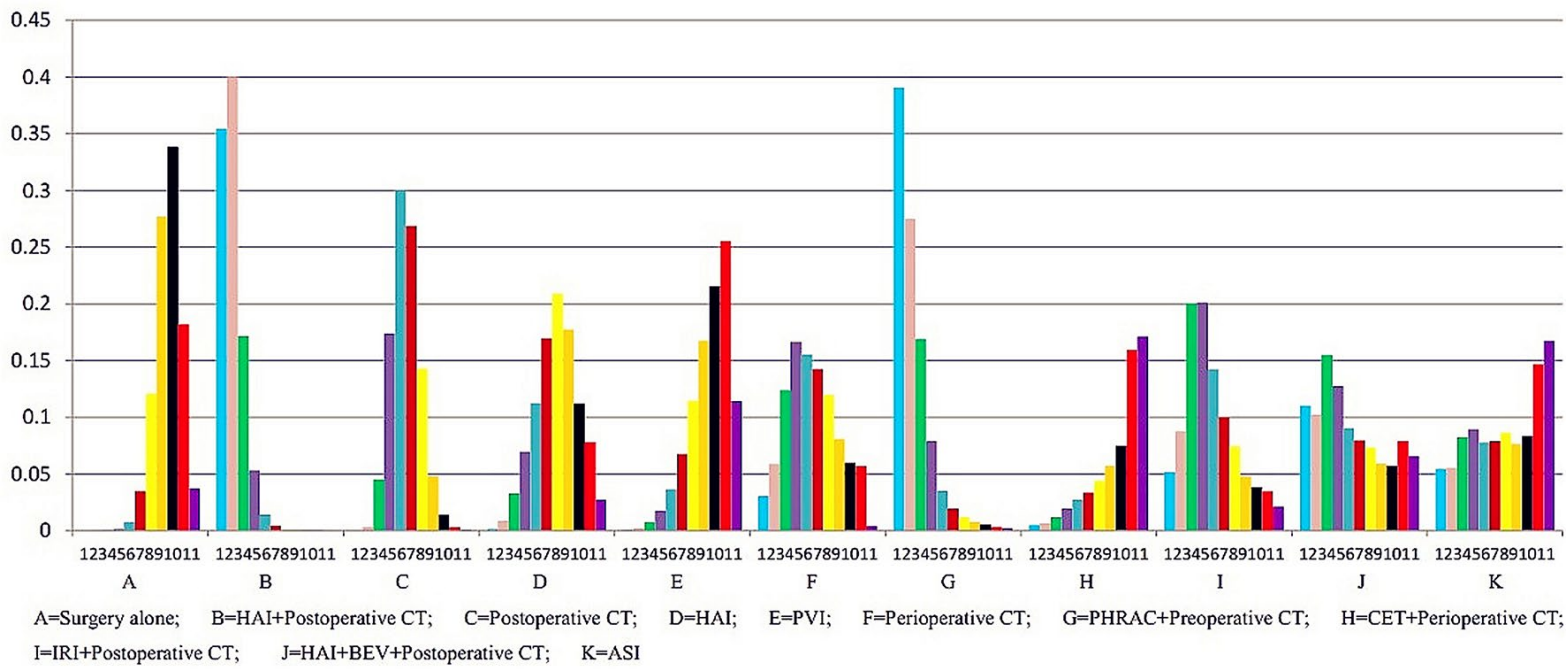
Ranking of treatments in disease-free survival.

The results of NMA are presented in **Table 3**. Three treatments that reached statistical significance in terms of PFS compared with surgery alone were HAI plus postoperative CT [HR = 1.44 with 95% CI: (1.19–1.75)], postoperative CT [HR = 1.14 with 95% CI: (1.01–1.29)], and PHRAC plus preoperative CT [HR = 1.41 with 95% CI: (1.03–1.89)]. However, perioperative CT was not associated with a statistically significant survival advantage compared with surgery alone [HR = 1.15 with 95% Crl: (0.87–1.51). When the three treatments were compared with surgery alone, we found statistically significant differences between HAI plus postoperative CT and postoperative CT [HR = 0.80 with 95% CI: (0.67–0.95)]. In addition, there were statistically significant differences between PVI and PHRAC plus preoperative CT [HR = 1.56 with 95% CI: (1.08–2.17)], HAI plus postoperative CT and PVI [HR = 0.64 with 95% CI: (0.49–0.84)], and HAI plus postoperative CT and HAI [HR = 0.75 with 95% Crl: (0.59–0.96)]. The results of the rank probabilities of the 11 treatments in DFS are summarized in **Figure 6**, suggesting that PHRAC plus preoperative CT exhibited the highest probability of benefitting DFS, followed by HAI plus postoperative CT and the combination of HAI and BEV plus postoperative CT.

### Choice of Model—Fixed Effect or Random Effect

The DIC was -5.256 for the fixed effect model and -13.69 for the random effect model. The posterior mean of the residual deviance for the fixed effect model was greater than the random effect, at 17.53 vs. 10.98. The heterogeneity was 40.6% between HAI plus postoperative CT and surgery alone for OS. These outcomes show that the random effect model produced the best fit for the data and suggest consistency within the model.

### Quality of Evidence

**Figure 7:**
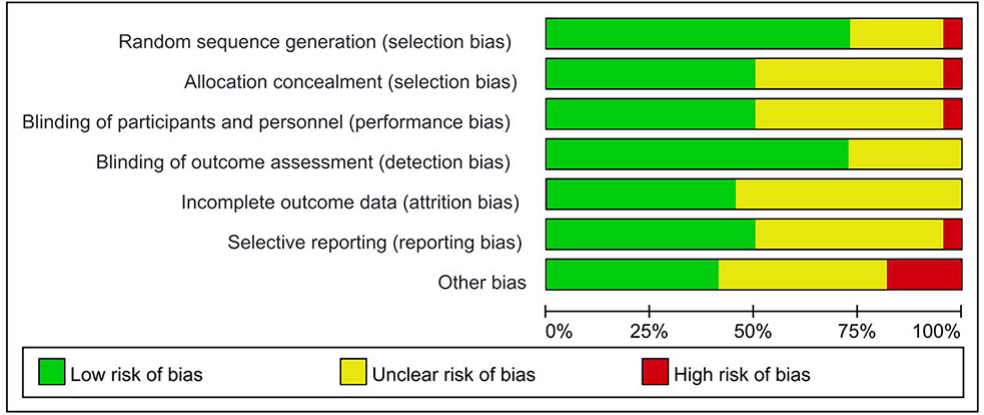
Risk of bias graph for all studies included.

**Figure 8:**
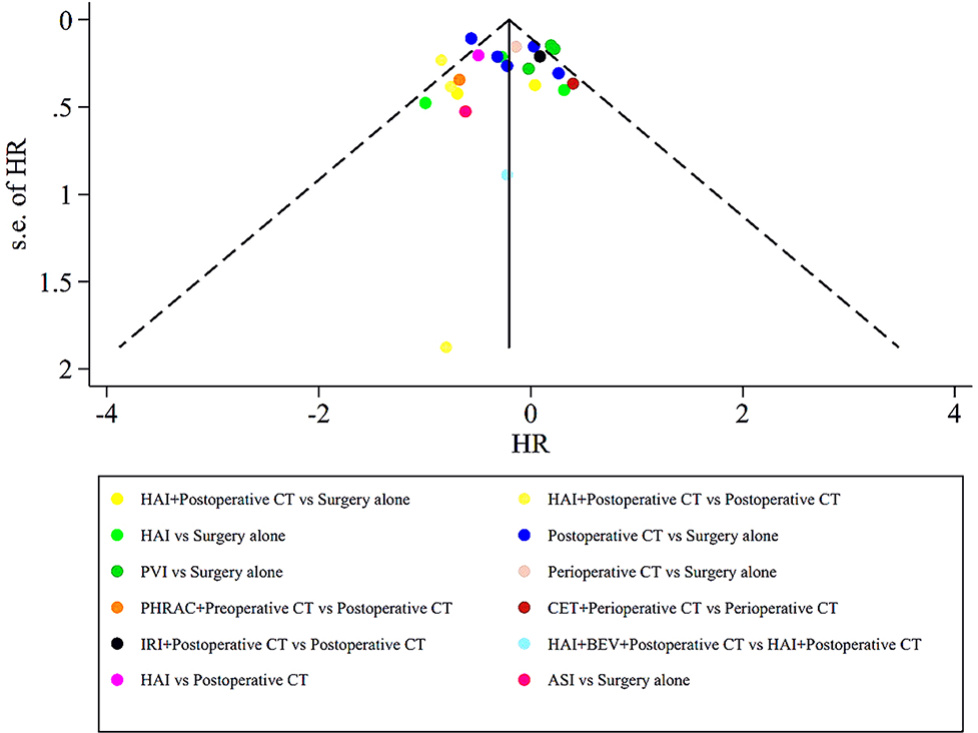
Results of the comparison-adjusted funnel plots.

The assessment of the risk of bias for eligible RCTs included in the NMA is presented in **Figure 7** and **Table 1**, according to the Cochrane risk-of-bias tool and the RoB 2.0 tool, suggesting no severe risk of bias. The heterogeneity of the pairwise comparison between the two surgical procedures is presented in **Tables 2** and **4**, suggesting no significant heterogeneity, or not assessable for most direct comparisons, except for “HAI vs. Surgery” (I^2^ = 65.9%) and “Postoperative CT vs. Surgery alone” (I^2^ = 71.1%). The results of the comparison-adjusted funnel plots are presented in **Figure 8**, which did not reveal any evidence of apparent asymmetry, indicating no significant publication bias.

### Sensitivity and Subgroup Analysis

**Figure 9:**
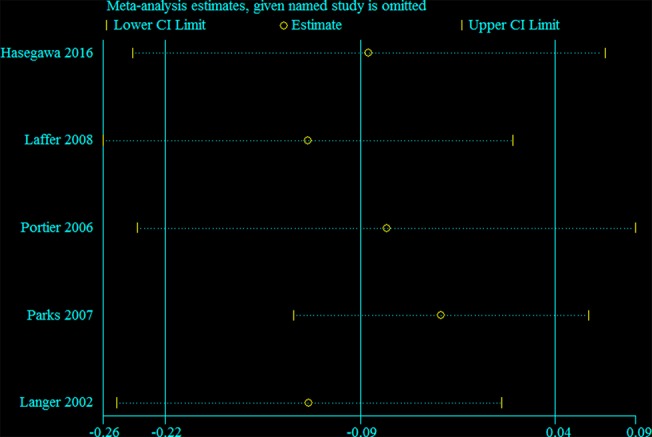
Sensitivity analysis of group with large heterogeneity.

**Figure 10:**
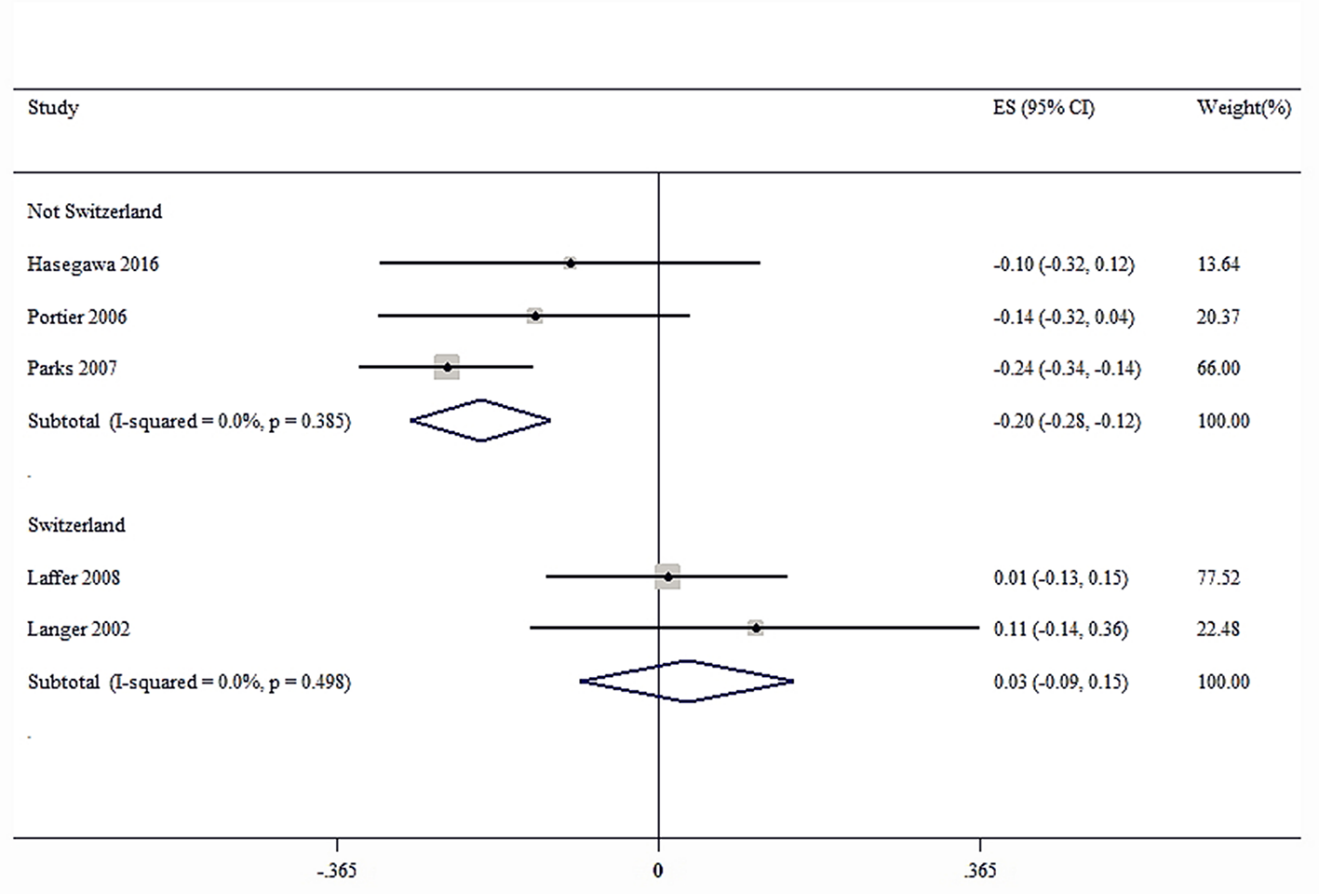
Subgroup analysis of group with large heterogeneity.

Sensitivity analysis was performed for the group with large heterogeneity, which suggested that the included study estimates were basically within the CI of the total effect values, indicating that the results were relatively stable (**Figure 9**). At the same time, subgroup analysis was used for the included studies grouped by country, which suggested that the heterogeneity disappeared, indicating that different countries may be sources of heterogeneity (**Figure 10**). This may be related to genetic and environmental factors in different countries.

## Discussion

The present systematic review is the first, to our knowledge, to compare the 11 approaches available for the treatment of the resectable CRLM by using NMA. This NMA was combined direct and indirect evidence from 22 RCTs including 6,115 patients with operable CRLM to estimate the relative efficacy of the 11 treatment strategies for outcomes involving OS and DFS.

The results demonstrated that HAI plus postoperative CT provide survival benefits compared with surgery, which was consistent with the results of a previous study (Kemeny et al., 2002). In addition, HAI plus postoperative CT showed a significant increase in OS compared with postoperative CT [HR = 1.27 with 95% Crl: (1.02–1.58)], which was in accord with a contemporaneous study conducted at the Memorial Sloan Kettering Cancer Center for patients with resected colorectal hepatic metastases (Kemeny et al., 1999). After hepatectomy, the 2-year survival rates of HAI plus postoperative CT and postoperative CT were 86 and 72%, respectively. However, PHRAC plus preoperative CT and perioperative CT was not associated with a statistically significant survival advantage compared with surgery alone [HR = 0.94 with 95% Crl: (0.68–1.30); HR = 0.70 with 95% Crl: (0.50–1.03), respectively]. Similarly, a previous study showed that there was no difference in OS with the addition of perioperative CT with FOLFOX4 compared with surgery alone in patients with resectable liver metastases from CRC (Nordlinger et al., 2013). The results of rank probabilities showed that the combination of HAI and BEV plus postoperative CT exhibited the highest probability of being the best treatment for improving OS in people with resectable CRLM, followed by PHRAC plus preoperative CT, and HAI plus postoperative CT. A pooled analysis of cohorts of older patients with CRLM from two randomized clinical trials conducted by Kabbinavar et al. (Kabbinavar et al., 2009) indicates that BEV plus postoperative CT improved OS and PFS, similar to the benefits observed in younger patients. Additionally, the risks of treatment did not increase compared with younger patients. Accordingly, Hurwitz et al. (Hurwitz et al., 2004) demonstrated that BEV plus postoperative CT results in a statistically significant improvement in survival for patients with CRLM. Multiple randomized trials have demonstrated *that the* humanized monoclonal vascular endothelial growth factor (VEGF) antibody BEV can significantly improve the prognoses of first- and second-line CT in patients with CRLM (Kabbinavar et al., 2003; Kabbinavar et al., 2005; Saltz et al., 2008). In addition, CET is a monoclonal antibody directed against the epidermal growth factor receptor, which can improve OS and PFS in patients with CRC and maintain a quality of life for patients (Jonker et al., 2007). Oki et al. (Oki et al., 2019) believed that liver metastases are less than or equal to 4 and larger than 5 cm in diameter, which is a good indicator for the use of CET for initially unresectable CRLM. However, Karapetis et al. (Karapetis et al., 2008) showed that patients with CRC bearing wild-type KRAS did benefit from CET, whereas those bearing mutated KRAS did not. Van et al. (Van Cutsem et al., 2009) also demonstrated that first-line treatment with CET plus IRI, FU, and LV (FOLFIRI) can reduce the risk of MCRC progression in patients with KRAS wild-type tumors. Primrose et al. (Primrose et al., 2014) showed that combination of CET and CT plus surgery for operable CRLMs in KRAS exon 2 wild-type patients leads to shorter PFS; therefore, CET in combination with CT cannot be recommended for patients with operable CRLMs.

When we focused on the results for PFS, three approaches that led to a significant improvement in DFS compared with surgery alone were HAI plus postoperative CT [HR = 1.44 with 95% Crl: (1.19, 1.75)], postoperative CT [HR = 1.14 with 95% Crl: (1.01, 1.29)], and PHRAC plus preoperative CT [HR = 1.41 with 95% Crl: (1.03, 1.89)], which were in accord with the results of several previous studies (Langer et al., 2002; Parks et al., 2007; Laffer et al., 2008). *Additionally*, in the European Organisation for Research and Treatment of Cancer study (Nordlinger et al., 2008), perioperative CT increased PFS versus no CT (with PFS in the treated arm that underwent liver resection of 42 and 28% at 3 and 5 years, respectively). In addition, PHRAC plus preoperative CT significantly improved DFS when compared with PVI [HR = 1.56 with 95% CI: (1.08–2.17)]. PHRAC, which consists of regional arterial CT and hepatic arterial CT, is one of the neoadjuvant CT methods, which has been proven to improve survival (Xu et al., 2007). The results of rank probabilities demonstrated that PHRAC plus preoperative CT might be the best treatment for improving DFS in people with resectable CRLM, followed by HAI plus postoperative CT and the combination of HAI and BEV plus postoperative CT.

The liver is known to have a double blood supply. The blood supply to liver metastases is mainly from the HA, while the normal hepatocytes are mainly from the portal vein. Since the residual tumors after hepatectomy could have a diameter of 2 to 3 mm, most of their blood supply is likely to come from the HA (Ackerman, 1974). Injection of CT directly into the HA not only increases local drug concentration and reduces systemic response but also preserves blood supply to normal liver tissue. Postoperative CT can completely kill the remaining cancer cells in the body and prevent distant metastasis. BEV specifically blocks VEGF, inhibits the formation of new blood vessels, and destroys existing neovascularization network, so as to normalize tumor blood vessels and facilitate the release of CT drugs into tumor to play its cell-killing role; BEV can also inhibit the complementation of endothelial stem cells, improve the environment of tumor hypoxia, to reduce the stimulation of VEGF secretion, thereby inhibiting the formation of new blood vessels (Ferrara et al., 2003). It is worth mentioning that the combination of HAI and BEV plus postoperative CT needs to consider the toxic and side effects of the drug, so the control of dosage and timing becomes particularly important.

In addition, many studies (Ertel et al., 1993; Termeer et al., 2000; Washburn and Schirrmacher, 2002) have shown that infection of tumor cells with live NDV results in efficient upregulation of MHC class I and cell adhesion molecules on the surface of tumor cells, and leads to an improved tumor cell/T cell interaction and increased T cell co-stimulatory activity. Meanwhile, Zeng et al. (Zeng et al., 2002) showed that NDV can induce the production of various cytokines, such as interferons and chemokines, which affect the migration, activation, and cytotoxic activity of various immune cells. Finally, Schulze et al. (Schulze et al., 2009) have shown that ASI with an autologous tumor cell vaccine-NDV in colon cancer patients appears to be beneficial for prolonging overall and metastasis-free survival. However, the small number of participants in this study reduced the statistical power and thus limited the generalization of the results. Therefore, further clinical research is needed.

## Limitations

This NMA is acknowledged to have several limitations. First, except for “HAI vs. Surgery” and “Postoperative CT vs. Surgery alone,” most of the pairwise comparisons lack significant heterogeneity, which may be because the applied protocols are different from the past. Second, we did not examine the distribution of methodological and clinical variables in detail, which may provide potential sources of either heterogeneity or inconsistency in every comparison of specific groups of trials, although our pooled estimates were performed in random effect mode. Third, important conference abstracts were not included in our study. *Finally, the current study was not registered, and there may be a small bias, but we still follow the steps of the systematic review strictly*.

## Conclusion

The present study is the first to compare the 11 treatment strategies available for the treatment of resectable CRLM by using NMA, and the results demonstrated that the combination of HAI and BEV plus postoperative CT exhibited the greatest odds of being the most effective treatment for improving OS, and PHRAC plus preoperative CT showed the greatest odds of improving DFS. Nevertheless, large prospective studies are required to investigate the optimal neoadjuvant or adjuvant CT treatment for operable CRLM.

## Author Contributions

ZZh and CH designed the study. HL and CH screened studies and extracted data. Disagreements were resolved by discussion with JH. CH performed the statistical analyses. ZZh, JH, HL, ZZo, and CH reviewed the results, interpreted the data, and wrote the manuscript. All authors read and approved the final version of the paper.

## Funding

This present study was supported by the Program of National Natural Science Foundation of China (No.81560389; No.81860433), and the Natural Science Youth Foundation of Jiangxi Province (No.20192BAB215036).

## Conflict of Interest Statement

The authors declare that the research was conducted in the absence of any commercial or financial relationships that could be construed as a potential conflict of interest.
